# Mitochondrial Involvement and Impact in Aging Skeletal Muscle

**DOI:** 10.3389/fnagi.2014.00211

**Published:** 2014-09-10

**Authors:** Russell T. Hepple

**Affiliations:** ^1^Department of Kinesiology, McGill University Health Center, McGill University, Montreal, QC, Canada

**Keywords:** sarcopenia, bioenergetics, mitochondria, skeletal muscle, reactive oxygen species, permeability transition, oxidative stress, muscle atrophy

## Abstract

Atrophy is a defining feature of aging skeletal muscle that contributes to progressive weakness and an increased risk of mobility impairment, falls, and physical frailty in very advanced age. Amongst the most frequently implicated mechanisms of aging muscle atrophy is mitochondrial dysfunction. Recent studies employing methods that are well-suited to interrogating intrinsic mitochondrial function find that mitochondrial respiration and reactive oxygen species emission changes are inconsistent between aging rat muscles undergoing atrophy and appear normal in human skeletal muscle from septuagenarian physically active subjects. On the other hand, a sensitization to permeability transition seems to be a general property of atrophying muscle with aging and this effect is even seen in atrophying muscle from physically active septuagenarian subjects. In addition to this intrinsic alteration in mitochondrial function, factors extrinsic to the mitochondria may also modulate mitochondrial function in aging muscle. In particular, recent evidence implicates oxidative stress in the aging *milieu* as a factor that depresses respiratory function *in vivo* (an effect that is not present *ex vivo*). Furthermore, in very advanced age, not only does muscle atrophy become more severe and clinically relevant in terms of its impact, but also there is evidence that this is driven by an accumulation of severely atrophied denervated myofibers. As denervation can itself modulate mitochondrial function and recruit mitochondrial-mediated atrophy pathways, future investigations need to address the degree to which skeletal muscle mitochondrial alterations in very advanced age are a consequence of denervation, rather than a primary organelle defect, to refine our understanding of the relevance of mitochondria as a therapeutic target at this more advanced age.

## Introduction

Progressive atrophy is a defining feature of aging skeletal muscle and when it becomes severe in very advanced age (≥80 years of age), it can lead to weakness that precipitates mobility impairment, an increased risk of falls, and physically frailty (Cruz-Jentoft et al., 2010). For this reason, understanding the mechanisms underlying aging muscle atrophy so that suitable therapeutic targets can be identified is key to promoting health and mobility in the elderly. Whilst many possible targets have been suggested, the most effective therapeutic targets will be those that serve as a nexus point for modulating a wide range of cellular functions that are affected with aging; a concept that brings to mind the role of the mitochondrion.

**Figure 1:**
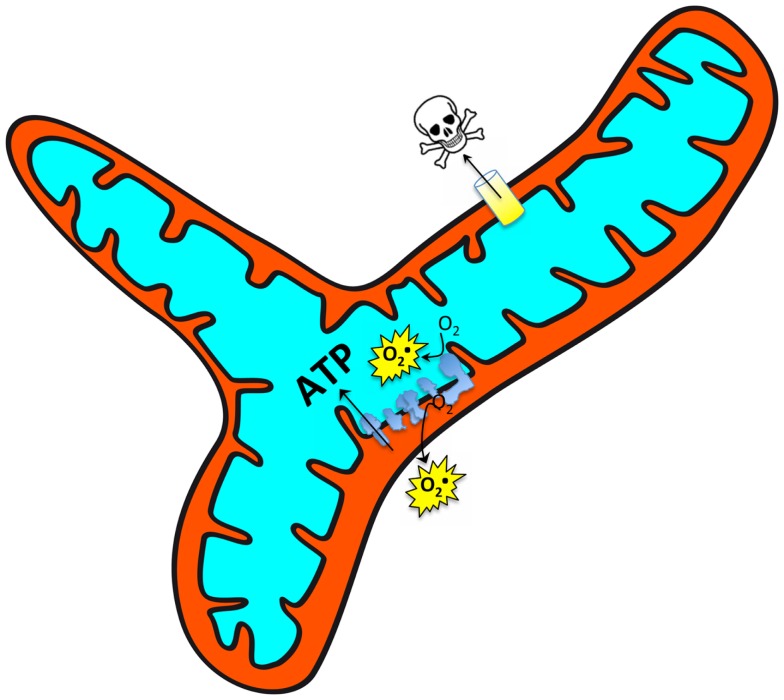
**Mitochondria respond to a wide array of intracellular signals and accordingly modulate their function [ATP production, reactive oxygen species (ROS) production, and sensitivity to permeability transition] across a large dynamic range to meet the cellular needs**. It is thus important to understand whether alterations observed in aging muscle represent a primary organelle defect versus a secondary (potentially adaptive) response to a changing cellular environment.

Mitochondria serve a central role as an integrator of a variety of signals within the cell, and accordingly vary their function to modulate energy supply, reactive oxygen species (ROS) signaling, and intrinsic pathways of apoptosis (Figure 1). For this reason, mitochondria have been frequently studied as a target for combating cellular aging. This is also true of muscle cells where one of the first studies to suggest impaired mitochondrial function may be involved in muscle aging was performed in flies and poignantly found that mitochondrial dysfunction was associated with loss of the wings (Rockstein and Brandt, 1963)! A great many studies have followed and although a substantial number have found evidence for impaired mitochondrial function in aging muscle (Rumsey et al., 1987; Trounce et al., 1989; Cooper et al., 1992; Conley et al., 2000; Gouspillou et al., 2010, 2014a,b; Picard et al., 2011a), others have not (Kent-Braun and Ng, 2000; Rasmussen et al., 2003; Lanza et al., 2005), underscoring the complexity of understanding in this area. The purpose of this review, therefore, is to address the basis for these complexities, identify relevant mechanisms therein, and finally to provide some suggestions for future investigation.

## Addressing the Question of Mitochondrial Dysfunction in Aging Muscle

There are numerous issues that need to be considered when evaluating the involvement of mitochondria in aging of skeletal muscle. The first issue to consider is the means by which the mitochondrial function is interrogated. For example, direct interrogation of mitochondrial function frequently employs mechanical isolation of mitochondria, a method that itself can induce alterations in the intrinsic function of the organelle (Picard et al., 2011c). Indeed, mechanical isolation of mitochondria not only exaggerates the magnitude of age-related impairment in various aspects of mitochondrial function but also induces changes not seen in a preparation where mitochondrial structure is preserved (Picard et al., 2010).

A second issue to consider is which functional outputs are quantified as it relates to the different roles that mitochondria serve in skeletal muscle, including energy provision (respiration), signaling (ROS emission), and regulation of apoptosis (e.g., through permeability transition and release of mitochondrial-derived apoptotic factors) (Picard et al., 2011a). Further to this point, it is also essential to account for the fact that some mitochondrial functional properties vary between fast twitch and slow twitch muscles (Picard et al., 2012). For example, slow twitch muscles generally exhibit lower mitochondrial ROS emission (Anderson and Neufer, 2006; Picard et al., 2008) and a lower capacity for calcium retention before permeability transition occurs (Picard et al., 2008) when compared to fast twitch muscles. Thus, in situations where muscle exhibits a shift in fiber type with aging, the impact of this shift on the mitochondrial function properties needs to be considered.

The third issue to consider is the age studied and in this respect, it is important to address mitochondrial function across the aging continuum, including study of ages that are most affected by the consequences of aging muscle atrophy. As noted in the section “Introduction,” this is typically ≥80 years of age (Cruz-Jentoft et al., 2010) and thus far there is very little known about muscle mitochondrial alterations in this age group from human studies, although several studies in the animal literature exist (Chabi et al., 2008; Ljubicic et al., 2009; Joseph et al., 2010; Picard et al., 2011a). Related to this point, when using an animal model system the age studied should represent points along the trajectory of muscle aging that are relevant for human muscle.

A fourth issue to consider is that of physical activity because it is well-established that skeletal muscle mitochondrial content is highly adaptable and not only increases in response to elevated metabolic demand but also decreases in response to reduced metabolic demand, with the latter being the typical situation in aging (Martin et al., 2014). As such, understanding the degree to which mitochondrial content and functional alterations are an obligatory consequence of aging versus being wholly or partially avoidable through a physically active lifestyle, is of critical importance.

Finally, since mitochondria serve as central integrators of a wide variety of cellular signals and thus exhibit a wide range of what should be considered physiological (rather than pathological) function (Picard et al., 2011b), it is essential to consider the involvement of cellular alterations that are extrinsic to the mitochondria as modulating factors in their function before concluding that a primary mitochondrial defect exists. For example, aging in muscle has long been known to induce neurological alterations such as neuromuscular junction instability leading to denervation (Tomonaga, 1977; Oda, 1984; Edstrom et al., 2007; Rudolf et al., 2014), and these changes may themselves exert an impact on the cellular environment that modulates mitochondrial function (Csukly et al., 2006; Muller et al., 2007; Bhattacharya et al., 2009). The suitability of the mitochondrion as a primary therapeutic target in this scenario needs to be carefully considered as reversing mitochondrial alterations that are secondary to denervation [e.g., those that result in activation of atrophy pathways (Romanello et al., 2010; Rowan et al., 2012)] could make the situation worse rather than better by preventing atrophy of denervated fibers and thereby increasing the burden on the remaining muscle fibers. These issues will be addressed in detail in the sections that follow to inform our current understanding of the role played by mitochondrial dysfunction in aging muscle atrophy and the resulting implications for intervening at this level.

## Methods for Evaluating Mitochondrial Function

Many different methodological approaches have been taken to evaluate mitochondrial function in aging muscle, including direct measures (e.g., isolated mitochondria, permeabilized myofibers) and indirect measures [e.g., phosphocreatine (PCr) recovery kinetics following muscle contractions, enzyme assays, protein content, etc.]. Similarly, a variety of functional outputs of mitochondria have been examined, with indices related to mitochondrial respiration being by far the most frequent and measures of ROS emission second most frequent, whereas to date there are relatively few studies examining mitochondrial sensitivity to an apoptotic challenge, particularly in the human literature. Importantly, the impact of aging on the different aspects of mitochondrial function is often quite variable from one function to the next and this has implications for understanding the involvement of mitochondria in aging muscle. For example, a reduced respiratory capacity could impact muscle fatigue by limiting energy provision to the myocyte (Stary et al., 2004; Chabi et al., 2008), an increase in ROS emission could induce cellular and organelle oxidative stress and thereby increase the requirement for removal of resulting damage (Fulle et al., 2004; Mansouri et al., 2006), and an increased susceptibility to permeability transition could increase the release of mitochondrial-localized apoptotic factors thereby contributing to nuclear loss and myocyte atrophy (Marzetti et al., 2010; Gouspillou et al., 2014b).

### Mitochondrial enzyme activities

By far the most common approach to gaining insights about mitochondrial function in aging muscle is the use of enzymatic assays of representative mitochondrial enzymes, such as citrate synthase and electron transport chain complexes, and these measures provide indirect insights into the energy producing (respiratory) capacity of the mitochondria. Most of the studies performed to date indicate a marked decline in mitochondrial enzyme activities from aging muscle (Bass et al., 1975; Lezza et al., 1994; Desai et al., 1996; Hagen et al., 2004), although some studies find this to be maintained (Orlander et al., 1978; Barrientos et al., 1996) and others report it to be highly variable from one muscle to the next (Houmard et al., 1998; Lyons et al., 2006). Importantly, individual enzyme activities do not provide unambiguous insights about the respiratory function of the intact mitochondrion because individual enzyme activities can become very disparately related to whole organelle function when the mitochondrion becomes defective. Thus, more direct and integrated measures of function are required to interpret aging impact.

### Mechanically isolated mitochondria versus saponin-permeabilized myocytes

**Figure 2:**
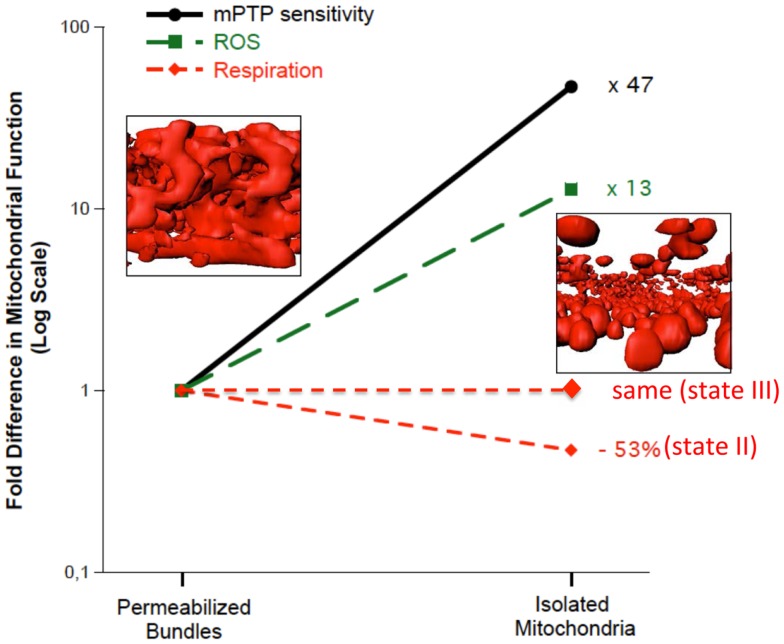
**Multiple features of mitochondrial structure and function are altered in mechanically isolated organelles compared to saponin-permeabilized myofibers (representing a preparation where all mitochondria are represented and their structure remains intact)**. In particular, the irregular tubular structure is lost following isolation, producing more homogenous spherical structures. Accompanying these structural changes is a marked potentiation of reactive oxygen species (ROS) emission and sensitization to mitochondrial permeability transition pore (mPTP) opening. Data are re-plotted from Picard et al. (2011c).

**Figure 3:**
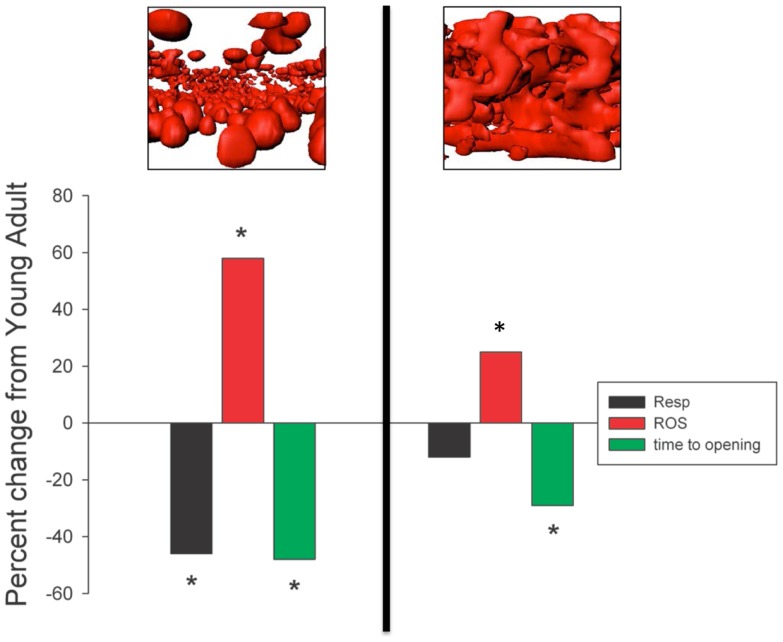
**Mechanical isolation (to the left of the vertical line) markedly exaggerates the apparent impact of aging on skeletal muscle mitochondria respiratory function, reactive oxygen species (ROS) emission, and sensitivity to mitochondrial permeability transition pore (mPTP) opening (permeability transition) compared to saponin-permeabilized myofibers (to the right of the vertical line)**. *P < 0.05 versus SEN. Data are re-plotted from Picard et al. (2010).

**Figure 4:**
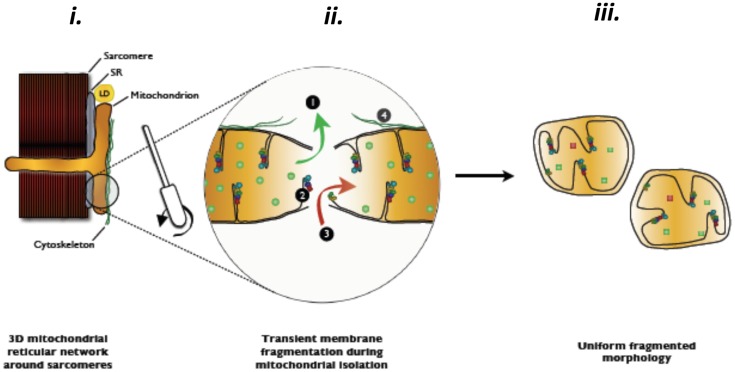
**Hypothesized contamination and/or matrix dilution occurring with the transient rupture of mitochondrial membranes during mitochondrial isolation procedures**. Reproduced with permission from Picard et al. (2011b).

Amongst the most frequently used methodological approaches to directly interrogate mitochondrial function involves the mechanical isolation of mitochondria (Chance and Williams, 1956). Although this method has been very widely used in the aging literature (Trounce et al., 1989; Boffoli et al., 1994; Capel et al., 2005; Chabi et al., 2008; Gouspillou et al., 2010), as we have recently reviewed (Picard et al., 2011b), not only does mechanical isolation markedly disrupt the normal architecture of skeletal muscle mitochondria from an irregular tubular network into smaller, relatively homogenous, spherical structures, but it also profoundly potentiates both mitochondrial ROS emission and sensitivity to permeability transition (Figure 2). The importance of this to studies in aging is illustrated by the fact that when we compared the apparent effect of aging on mitochondrial function in mitochondria isolated from skeletal muscle versus saponin-permeabilized myofibers (where mitochondrial structure is preserved) in very old rat muscle (35% survival rate, which is a similar relative age to ≥80 years old humans), we found the isolated mitochondria profoundly exaggerated the impact of aging (Picard et al., 2010). Specifically, with aging the decline in maximal mitochondrial respiratory capacity was fourfold greater, the increase in ROS emission was twofold greater and the reduction in time to permeability transition was twofold greater when examined in isolated mitochondria versus saponin-permeabilized myofibers (Figure 3). Not only this, but there were significant alterations in mitochondrial enzyme activity stoichiometry (reduced ratio of cytochrome oxidase to citrate synthase activity) and stoichiometry of respiratory states (suggestive of a defect in complex I) in isolated mitochondria with aging that were not seen in saponin-permeabilized myofibers. We have suggested that these latter observations may be indicative of a lesser ability of aged mitochondria to reseal during isolation procedures, since isolation induces transient disruption of tubular mitochondrial structures and subsequent reconstitution into the spherical organelles typical of isolates, resulting in greater contamination and/or loss of mitochondrial matrix constituents in isolates prepared from aged muscles (Picard et al., 2010) (Figure 4). Collectively, therefore, our data show that the method used to interrogate mitochondrial function can have a profound influence on the degree to which mitochondrial function appears to be altered in aging muscle and that generally speaking, the degree to which mitochondrial function is altered in aging muscle is considerably less severe than has often been considered. This notion is consistent with other studies that have used the saponin-permeabilized myofibers method, which have found relatively mild (Joseph et al., 2012) or in other cases no impairments in mitochondrial respiratory capacity in aging muscle (Tonkonogi et al., 2000; Hutter et al., 2007; Gouspillou et al., 2014b).

**Figure 5:**
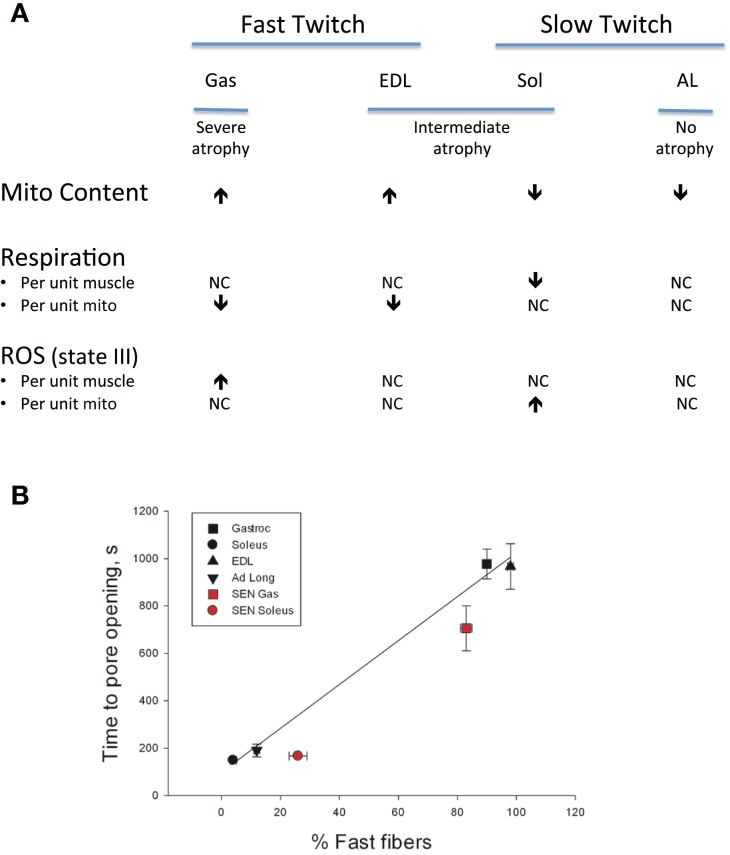
**Impact of aging on mitochondrial function in saponin- permeabilized myofibers prepared from rat muscles of contrasting fiber type and atrophy susceptibility is shown**. Gas = gastrocnemius muscle, EDL = extensor digitorum longus muscle, Sol = soleus muscle, AL = adductor longus muscle. **(A)** Impact of aging on mitochondrial content, respiration, and reactive oxygen species (ROS) emission responses. **(B)** Sensitivity of mitochondria to permeability transition following a calcium challenge and how this varies as a function of fast twitch fiber abundance. Solid black symbols represent the responses of young adult muscles, whereas the solid red symbols represent responses of senescent muscles. After taking into account the shift in proportion of fibers positive for fast myosin heavy chain with aging in each muscle, both the soleus and gastrocnemius muscles exhibit a sensitization to permeability transition. Data for mitochondrial content, respiration, ROS, and time to pore opening are taken from Picard et al. (2011a). Data for proportion of fibers positive for fast myosin heavy chain are taken from Carter et al. (2010).

Notwithstanding the point about mitochondrial respiratory alterations in aging muscle being less severe than often considered, in a follow-up study where we compared indices of mitochondrial function in saponin-permeabilized myofibers prepared from four rat muscles that exhibited contrasting fiber type and contrasting atrophy susceptibility with aging, we did find mitochondrial alterations that we believe bear consideration as a contributing factor in atrophy of aging muscle (Picard et al., 2011a). Our comparison involved two fast twitch muscles [extensor digitorum longus (EDL), gastrocnemius (Gas)] and two slow twitch muscles [soleus (Sol), adductor longus (AL)], where the Gas exhibited the most severe atrophy with aging, the EDL and Sol were similarly affected, and most strikingly, the AL actually hypertrophied by nearly 50% and the mitochondrial function was largely preserved in this muscle. Although the factors accounting for this profoundly better adjustment to aging in the rat AL remain under study, the other muscles exhibited varying degrees of mitochondrial functional alteration with aging. In particular, the Sol was the only muscle to exhibit a reduction in muscle respiratory capacity (Figure 5A) and in this case it was secondary to reduced mitochondrial content. In contrast, although the muscle respiratory capacity was largely preserved in both of the fast muscles, these two muscles had higher levels of representative subunits of the mitochondrial electron transport complexes, suggesting that the intrinsic respiratory capacity of the mitochondrial electron transport system was impaired in these fast muscles with aging. Muscle ROS emission normalized for respiration (an index of the proportion of O_2_ consumption lost to ROS leak) demonstrated a general trend to be elevated in all four muscles under state II and state III conditions, although after normalizing for mitochondrial content only the Sol demonstrated an increase, suggesting an increase in intrinsic mitochondrial ROS emission in aging muscle is not a general feature of aging muscle (Figure 5A). On the other hand, the sensitivity to permeability transition was increased in the fast twitch muscles, suggesting greater apoptotic potential. Although we did not observe a change in this property in the Sol muscle, despite its marked atrophy with aging, as noted in Section “Addressing the Question of Mitochondrial Dysfunction in Aging Muscle,” the time to mitochondrial permeability transition pore (mPTP) opening (permeability transition) in response to a calcium challenge is typically lower in slow twitch muscle (and higher in fast twitch muscle). As the aged Sol is characterized by a striking increase in the fraction of fibers that express fast myosin heavy chain isoforms (Edstrom and Ulfhake, 2005; Snow et al., 2005; Carter et al., 2010), after taking this shift into account, the aged Sol should have a longer time to mPTP opening than observed, meaning it too exhibits a sensitization to permeability transition with aging. This is depicted in Figure 5B as a time to pore (mPTP) opening that falls below the line predicted by the relationship between time to mPTP opening and percentage of fibers positive for fast myosin heavy chain. Collectively, therefore, since all three muscles, which atrophied with aging (EDL, Gas, Sol) exhibited a reduced time to permeability transition, whereas the only muscle that did not atrophy (AL) was unaffected, our results reveal that a general property of aging muscles undergoing atrophy is an increased mitochondrial sensitivity to an apoptotic challenge. Taken in context with the abundance of evidence for elevated recruitment of mitochondrial-mediated pathways of apoptosis in aging muscle (Dirks and Leeuwenburgh, 2002; Leeuwenburgh et al., 2005; Siu et al., 2005; Chabi et al., 2008; Marzetti et al., 2008; Gouspillou et al., 2014b), this change in mitochondrial function in aging muscle may be a key to induction of myofiber atrophy.

### Non-invasive spectroscopic methods

The final methodological approach to be discussed in this review is the use of non-invasive spectroscopic techniques to interrogate mitochondrial function in aging muscle. Although the value of these measures is that they provide insights into the function of the organelle in its native environment, as will be discussed here, under some circumstances this can pose challenges in deciphering whether a primary mitochondrial defect exists.

The most common non-invasive spectroscopic approach for monitoring mitochondrial function *in vivo* involves ^31^Phosphorous spectroscopy to determine PCr recovery time following muscle contractions, as the rate of PCr recovery is proportional to mitochondrial respiration and content (Mahler, 1985; Meyer, 1988; Prompers et al., 2014). While this is the only way currently available to gain insights into mitochondrial energy production *in vivo*; it is, however, important to note that the rate of PCr resynthesis following muscle contractions also depends upon oxygen supply (Haseler et al., 1999). Thus, if muscle blood flow is at all reduced in aging muscle during the recovery from contractions, noting that some studies have observed lower blood flow during dynamic exercise in the elderly (Lawrenson et al., 2003; Poole et al., 2003), the resulting lower muscle oxygen delivery with aging is a confounding factor in the interpretation of reduced mitochondrial energetic capacity in the elderly based upon slower PCr recovery kinetics alone.

Keeping this point in mind, although several studies report a reduced rate of PCr recovery following muscle contractions in aging human gastrocnemius (McCully et al., 1993) and vastus lateralis muscles (Conley et al., 2000), this was not seen in aging human tibialis anterior muscle of subjects matched for physical activity levels to the young comparison group (Kent-Braun and Ng, 2000; Lanza et al., 2005). Although differences in physical activity status may be part of the explanation for the different results between studies (Kent-Braun and Ng, 2000), it may also relate to the aforementioned impact of oxygen delivery and/or reflect differences in the impact of aging on mitochondrial function in different muscles, as we have shown that occurs in aged rodents (Picard et al., 2011a) (see Mechanically Isolated Mitochondria versus Saponin-Permeabilized Myocytes).

Non-invasive spectroscopic methods have also been used to evaluate mitochondrial coupling in skeletal muscle *in vivo*. In particular, several studies have now combined measures of PCr resynthesis (to derive what has been termed “ATPmax”) with optical spectroscopic assessment of myoglobin and hemoglobin oxygen saturations (to derive intramuscular oxygen consumption) to yield a measure of coupling efficiency based upon the quotient of ATP turnover (derived from the PCr spectroscopy measures) and oxygen consumption (Amara et al., 2007). These studies have identified mild uncoupling of mitochondria in both mouse (Marcinek et al., 2005) and human (Amara et al., 2007) skeletal muscle. As discussed in the following section, mild uncoupling has also been reported in saponin-permeabilized myofibers from physically active septuagenarian humans (Gouspillou et al., 2014b). Since uncoupling of mitochondria has been argued as one strategy for reducing mitochondrial ROS emission and promoting healthy longevity (Speakman et al., 2004), the mild uncoupling observed in aged muscle may represent an adaptive response to keep ROS within physiological levels, rather than a defect *per se* (Amara et al., 2007).

## Mitochondrial Content and Function in Relation to Age and Physical Activity

Amongst the most controversial issues about mitochondrial impact in aging muscle is that of mitochondrial content. Whilst some studies have concluded that mitochondrial content is reduced in aging muscle (Kerner et al., 2001; Chabi et al., 2008), some find no change (Mathieu-Costello et al., 2005; Callahan et al., 2014; Gouspillou et al., 2014b; Konopka et al., 2014), and others find it to vary between muscles (Lyons et al., 2006; Picard et al., 2011a). As was the case with measuring mitochondrial function, many different approaches have been taken to characterize mitochondrial content in aging muscle, including mitochondrial marker enzymes (e.g., citrate synthase or cytochrome oxidase activities), select mitochondrial proteins (e.g., porin, electron transport system complex subunits), mitochondrial DNA (mtDNA) copy number, and the gold standard, electron microscopic quantitation of mitochondrial volume density. Although the wide variety of approaches to inferring mitochondrial content changes in aging muscle has no doubt contributed to the variability in interpretation herein, even in studies using the gold standard of electron microscopy significant variation has been observed. For example, in two studies examining aged rodent muscle (Mathieu-Costello et al., 2005) and aged human muscle (Callahan et al., 2014), there was no change in skeletal muscle mitochondrial content assessed by electron microscopy with aging, whereas another study in aged humans did observe a decrease with aging using the same method and examining the same muscle (*vastus lateralis* muscle) (Conley et al., 2000), underscoring the discrepancy on the question of how aging impacts muscle mitochondrial content. Amongst the factors that contribute to this variability between studies, as noted in Section “Addressing the Question of Mitochondrial Dysfunction in Aging Muscle,” physical activity levels can play an important modulating role, where activity-matched subjects consistently show no decline in indices of mitochondrial content in aging muscle (Lanza et al., 2005; Gouspillou et al., 2014b). However, whether differences in physical activity status alone can explain the discordant results remains to be clarified.

Regardless of the controversy about the impact of aging on mitochondrial content, the capacity for generation of new mitochondria declines with aging at ages where the clinical consequences of aging in muscle are most relevant. Specifically, the ability of aged muscle to augment mitochondrial biogenesis in response to both endurance exercise training (Betik et al., 2009) and more severe muscle activity induced by chronic electrical stimulation (Ljubicic and Hood, 2009; Ljubicic et al., 2009) is severely compromised in rat muscle from animals at a similar relative age as humans ≥80 years of age. This impairment appears to be secondary to a failure to upregulate the machinery regulating mitochondrial biogenesis, including peroxisome proliferator activated receptor gamma coactivator-1 alpha (PGC-1α) (Betik et al., 2009), and an accelerated degradation of precursor proteins destined for import into mitochondria (Huang et al., 2010). To date, no therapeutic advances have been developed to restore the mitochondrial biogenesis response in very old muscle, designating this as an issue of high priority for future investigations.

**Figure 6:**
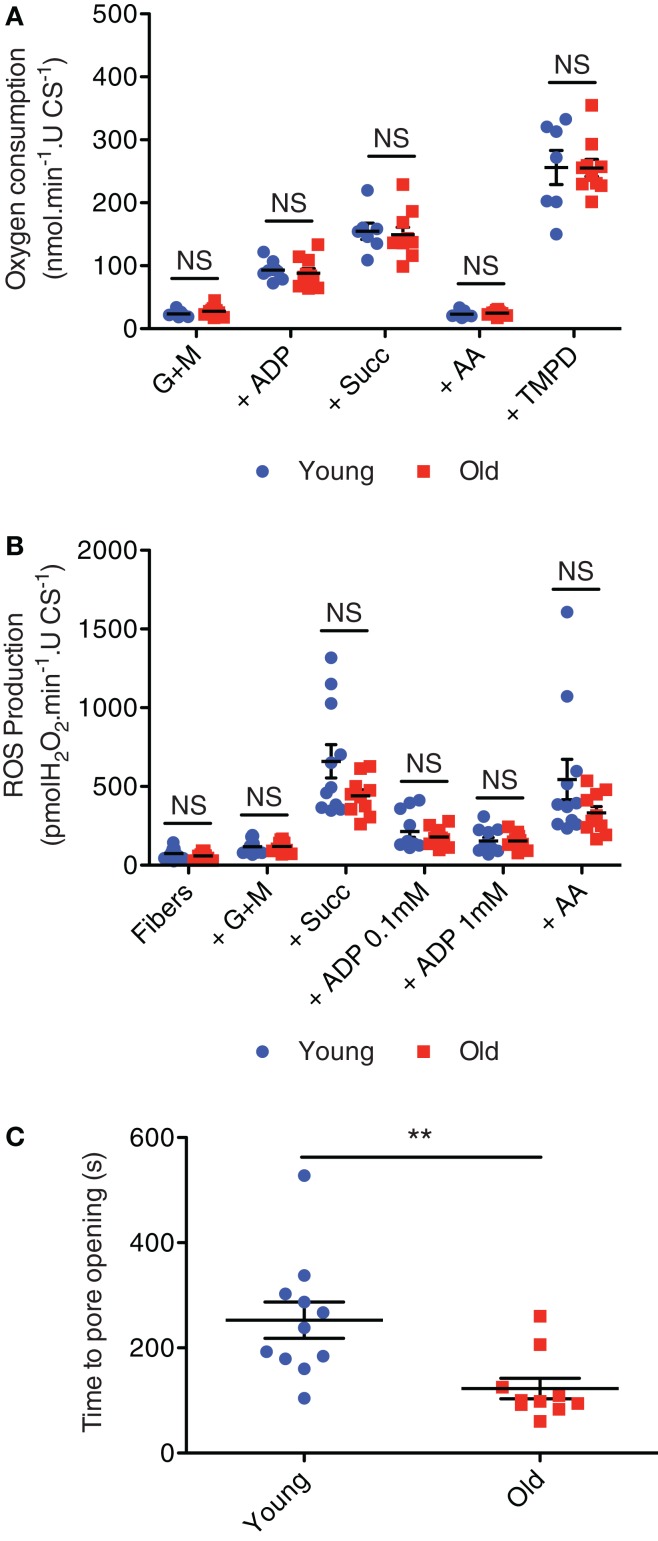
**Mitochondrial respiration (A), hydrogen peroxide emission (B), and sensitivity to permeability transition following a calcium challenge (C) in saponin-permeabilized myofibers prepared from vastus lateralis muscle biopsies obtained from recreationally active healthy young (24 ± 1 years; mean ± SE) and older (71 ± 2 years) men**. Figures are reproduced with permission from Gouspillou et al. (2014b). G + M = glutamate + malate; Succ = succinate; AA = antimycin A, TMPD = artificial electron donor. **P < 0.05 versus old.

Coming back to ages where muscle mitochondrial content remains adaptable (≤80 years of age), not only does physical activity level modulate mitochondrial content, but it can also modulate indices of mitochondrial function in aging muscle. In particular, previous studies have shown that when younger and older subjects are matched for physical activity levels, there is no reduction in muscle mitochondrial respiratory capacity with aging (Kent-Braun and Ng, 2000; Lanza et al., 2005; Safdar et al., 2010). We recently extended these results to show that, like respiratory capacity (Figure 6A), mitochondrial ROS emission is also not elevated with aging (Figure 6B) when comparing young adult and septuagenarian subjects with relatively high levels of recreational physical activity, although as seen in very old rodent muscle (see Mechanically Isolated Mitochondria versus Saponin-Permeabilized Myocytes; Picard et al., 2011a), we did observe a pronounced sensitization of the mitochondria to permeability transition (Gouspillou et al., 2014b) (Figure 6C). Indeed, the increased apoptotic sensitivity of aged mitochondria was also accompanied by a marked translocation of a mitochondrial-derived pro-apoptotic molecule, endonuclease G, to myonuclei in atrophied septuagenarian human muscle of recreationally active subjects, suggesting that this may be a key mechanism by which aged mitochondria contribute to aging muscle atrophy and one which cannot be circumvented through an active lifestyle (Gouspillou et al., 2014b). Potential mechanisms for this will be discussed in Section “Mechanisms of Primary Defects in Mitochondrial Function in Aging Muscle.”

## Mechanisms of Primary Defects in Mitochondrial Function in Aging Muscle

As noted above, a sensitization of mitochondria to permeability transition (Picard et al., 2011a) and release of mitochondrial-derived pro-apoptotic factors (Chabi et al., 2008; Marzetti et al., 2008) is evident in aged muscle, even in those who remain physically active (Gouspillou et al., 2014b). In accounting for the reasons for mitochondrial dysfunction in aging muscle, two primary hypotheses have been put forth to explain declining mitochondrial function with aging in general. The first hypothesis relates to the fact that mitochondria have their own genome, which, although only encoding for a small fraction of all mitochondrial proteins, is essential for production of normally functioning mitochondria. Since this genome accumulates damage progressively with aging (Richter et al., 1988; Katayama et al., 1991; Simonetti et al., 1992; Melov et al., 1995), it is posited that this leads to impaired synthesis of mitochondria and/or synthesis of mitochondria with aberrant function (Hiona and Leeuwenburgh, 2008). The second hypothesis relates to the fact that mitochondria must be regularly removed and replaced to preserve their fidelity (Schiavi and Ventura, 2014); it is posited that this process becomes impaired with aging, resulting in accumulation of damaged mitochondria with aberrant function (Terman et al., 2010; Joseph et al., 2013). The available evidence relating to these two hypotheses will be discussed in detail below.

### Significance of mtDNA mutation to mitochondrial dysfunction and aging muscle atrophy

The mtDNA molecule is a small circular genome (approximately 16 k base pairs in length) encoding 37 genes, of which 22 are for transfer RNAs, 2 are for ribosomal RNAs, and 13 are for polypeptide subunits of the respiratory chain and ATP synthase. In this last respect, the mtDNA encodes for 7 subunits of complex I, 1 subunit of complex III, 3 subunits of complex IV, and 2 subunits of the ATP synthase, and these components are essential to normal respiratory function. As evidence of the importance of mtDNA, there is marked mitochondrial dysfunction in patients with mtDNA mutations and when these mutations occur in skeletal muscle they can result in profound exercise intolerance (Taivassalo et al., 2003). As noted above, mtDNA mutations increase progressively with increasing age in multiple tissues, including skeletal muscle (Katayama et al., 1991; Melov et al., 1995; Bua et al., 2006). Furthermore, the focal accumulation of mtDNA mutations to high levels in skeletal myocytes with aging has been implicated in fiber atrophy, breakage, and loss (Lee et al., 1998; Wanagat et al., 2001; Bua et al., 2002). However, this latter aspect seems to be losing favor for several reasons. In particular, the proportion of fibers with severe mitochondrial dysfunction arising from mtDNA mutations is very low and these fibers do not seem to be consistently smaller than fibers with healthy mitochondria (Rowan et al., 2011). Further to this point, the proportion of fibers with focal atrophy at regions coinciding with high levels of mutant mtDNA and severe electron transport system dysfunction in human skeletal myocytes is also too low (in two subjects >65 years of age, only 5% of fibers with electron transport chain dysfunction exhibited focal atrophy; Bua et al., 2006) to be biologically meaningful compared to other causes of atrophy in aging muscle, particularly denervation, which is the primary cause of myofiber atrophy in very old rat muscle (Rowan et al., 2012).

Although focal accumulation of mtDNA mutations does not appear to be a primary cause of skeletal myocyte atrophy with aging, a mouse engineered with a faulty mtDNA proof-reading enzyme, the PolG mutant mouse, exhibits a dramatic increase in rate of mtDNA mutation, a markedly shortened lifespan, and numerous features that resemble premature aging (Trifunovic et al., 2004, 2005; Kujoth et al., 2005), including muscle atrophy (Hiona et al., 2010). Interestingly, the mitochondrial phenotypes observed in this so-called mtDNA mutator mouse differ in important ways from what is seen in normally aging muscle. In particular, whereas the PolG mutant mouse exhibits reduced levels of mitochondrial electron transport system complex subunits in skeletal muscle, as discussed in Sections “Mechanically Isolated Mitochondria versus Saponin-Permeabilized Myocytes” and “Mitochondrial Content and Function in Relation to Age and Physical Activity” this is not a consistent finding in aging rat (Picard et al., 2011a) or human muscle (Gouspillou et al., 2014b) where levels of mitochondrial electron transport system complex subunits are often higher in aged muscle. Similarly, whereas markers of mitochondrial fission and autophagy are higher in the PolG mouse muscle, normally aged mouse muscle exhibits higher levels of markers of mitochondrial fusion and lower levels of markers of autophagy (Joseph et al., 2013). On this basis, mtDNA mutations *per se* are unlikely to be the root cause of mitochondrial dysfunction in aging muscle.

### Impaired mitochondrial autophagy (mitophagy) in aging muscle

As mentioned above, mitochondria normally undergo degradation and replacement to ensure the fidelity of mitochondrial function. Indeed, impairment in mitochondrial autophagy (mitophagy) is implicated in a wide variety of neudegenerative disorders (Chu, 2010; Scheibye-Knudsen et al., 2014), in addition to normal aging (Schiavi and Ventura, 2014). Although in skeletal muscle the rate of mitochondrial turnover is unknown (nor it is understood if it differs between species), mitochondria have a half-life of approximately 2 days in mouse liver (Miwa et al., 2008), which when scaled up to the lifespan of an organism means that mitochondria undergo hundreds to thousands of cycles of turnover throughout the aging process. Clearly, therefore, if this rate of renewal were to decline, it seems a likely basis leading to accumulation of mitochondria with aberrant function in aging muscle.

Mitophagy is a tightly regulated process whereby dysfunctional/damaged mitochondria are targeted for removal by incorporation into autophagosomes for subsequent degradation by lysosomes. This targeting of dysfunctional mitochondria for removal depends upon several mechanisms that are likely relevant for aging. For example, mitophagy is elevated in response to mitochondrial fragmentation, reduced mitochondrial membrane potential (e.g., as occurs in mitochondria undergoing permeability transition), and increased mitochondrial ROS emission (Romanello et al., 2010; Twig and Shirihai, 2011; Schiavi and Ventura, 2014). Evidence that mitophagy is impaired in aging muscle is only now beginning to accumulate. Firstly, the fact that mitochondria with sensitization to permeability transition accumulate in aging muscle (Gouspillou et al., 2014b) is evidence that mitophagy of damaged mitochondria is impaired since permeability transition causes loss of mitochondrial membrane potential, a potent stimulus for mitophagy (Twig et al., 2008). In addition, two studies have now shown that Parkin, a mitochondrial-targeted ubiquitin ligase, which interacts with the autophagy protein LC3 to induce formation of autophagosomes around dysfunctional mitochondria, is reduced in skeletal muscle of both physically active septuagenarian men (Gouspillou et al., 2014b) and in physically inactive frail older women (Drummond et al., 2014). Furthermore, impairments in the signaling pathway that regulates mitochondrial quality control have also been reported in aging mouse muscle (Joseph et al., 2013) and aging human muscle (Koltai et al., 2012). Thus, whereas the significance of mtDNA mutations in mitochondrial dysfunction in aging muscle is less clear, impaired mitophagy appears a likely contributor to accumulation of dysfunctional mitochondria in aging muscle. Importantly, that this appears to also occur in physically active subjects (Gouspillou et al., 2014b) underscores that new therapeutic approaches will be required to address this problem.

Although their particular role in aging remains to be established, two promising new targets involved in regulating degradation of damaged mitochondria are the micro RNA miR137 and the AAA ATPase p97. miR137 has been identified to inhibit mitophagy occurring in response to hypoxia, through reducing the interaction of LC3 with two mitophagy receptors, FUNDC1 and NIX (Li et al., 2014). p97 is specifically involved in targeting and removal of carbonylated mitochondrial proteins for subsequent degradation by the ubiquitin proteasome (Hemion et al., 2014). If these pathways are disrupted in aging muscle, they are likely targets for improving removal of damaged mitochondria and thus, ameliorating aging impact.

## Extrinsic Factors That Could Modulate Mitochondrial Function in Aging Muscle

Although mitochondrial changes in aging muscle are most often considered as primary dysfunction suitable for therapeutic targeting, this view does not consider the influence of factors extrinsic to the mitochondrion that constitute the aging intracellular *milieu*. For example, age-related changes in the intracellular *milieu* could depress certain aspects of mitochondrial function independent of the intrinsic mitochondrial functional capacity *per se* and recent data support this idea. In addition, other changes impacting aging muscle, such as impaired neuromuscular junction stability and myofiber denervation, could also modulate mitochondrial function as a secondary consequence.

**Figure 7:**
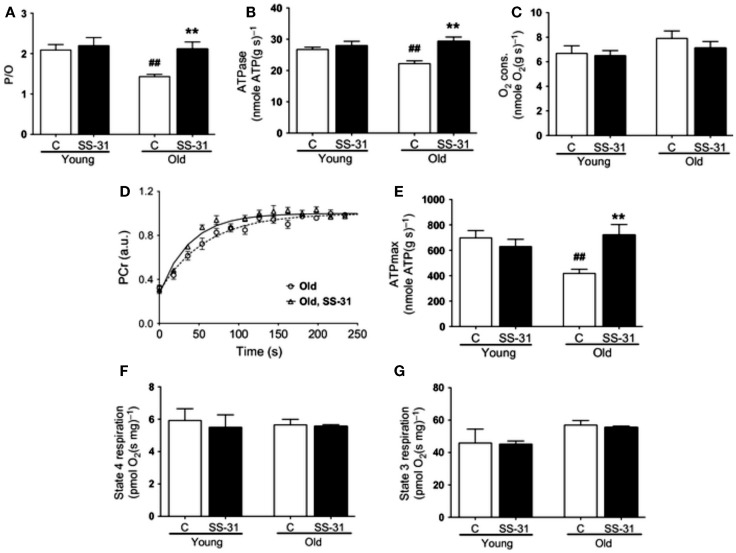
**Influence of the aging intracellular *milieu* on mitochondrial function *in vivo* in mouse skeletal muscle**. **(A)** P/O ratio, **(B)** *in vivo* ATPase rate; **(C)** *in vivo* rate of oxygen consumption; **(D)** *in vivo* rate of phosphocreatine (PCr) resynthesis following muscle contractions; **(E)** *in vivo* maximal rate of ATP synthesis (ATPmax); **(F)** state 4 respiration in saponin-permeabilized myofibers; **(G)** state 3 respiration in saponin-permeabilized myofibers. Figures are reproduced with permission from Siegel et al. (2013).

In addressing the potential involvement of the aging *milieu* as an inhibitory influence on mitochondrial function in aging muscle, Siegel et al. (2013) recently characterized mitochondrial function *in vivo* in distal hindlimb muscles of young (5 months old) and aged (27 months old) mice using optical and ^31^Phosphorous magnetic resonance spectroscopy, finding a decrease in mitochondrial respiratory function (reduced maximal ATP production rate, reduced coupling, and reduced rate of PCr resynthesis). Strikingly, these age-related impairments in mitochondrial respiratory function were restored to youthful levels 1 h following the administration of a mitochondria-specific antioxidant peptide, SS-31 (Figures 7A–E), and this was also accompanied by an increased muscle fatigue resistance and running time to exhaustion in the aged mice but not younger mice. In stark contrast to these acute *in vivo* results, when mitochondrial respiratory function was examined in saponin-permeabilized myofibers there was no detectable impairment in mitochondrial respiratory function with aging and administration of SS-31 had no effect (Figures 7F,G), despite inducing a reduction in mitochondrial ROS emission. The implications of these results is that, independent of the intrinsic function of the organelle, mitochondrial function can be impeded *in vivo* by oxidative stress in the intracellular environment of aging muscle. Thus, these recent findings underscore the importance of considering aspects of the aging *milieu* as contributing factors to impairing mitochondrial function *in vivo*, and demand further study of how this may affect other aspects of mitochondrial function (e.g., ROS emission, sensitivity to permeability transition) with aging. Furthermore, these results also show that manipulation of the aging *milieu*, rather than the mitochondrion directly, can be an effective strategy for improving mitochondrial function in aging muscle.

As noted in the section “Introduction,” mitochondria serve as cellular rheostats that integrate a wide variety of intracellular signals and modify aspects of their function as appropriate to those cellular conditions. Although intrinsic impairment in mitochondrial function involving an increased sensitivity to permeability transition in mitochondria from aged muscle has been demonstrated in both a sedentary rat model (Picard et al., 2011a) and in physically active aging humans (Gouspillou et al., 2014b), whether factors upstream of the mitochondrion can also modulate mitochondrial function is unclear. This question may be particularly relevant when aging muscle atrophy becomes severe and more likely to yield clinical impact because it is likely that additional mechanisms come into play in driving an acceleration of muscle atrophy. In this respect, as noted from the outset of this review, the age at which aging muscle atrophy is most likely to cause clinical consequences such as an increased risk of falls and physical frailty, is in individuals ≥80 years of age (Cruz-Jentoft et al., 2010). Significantly, in an aging rat model this corresponds to a period where muscle atrophy accelerates through the accumulation of small angular fibers characteristic of long-term denervation (Rowan et al., 2011).

**Figure 8:**
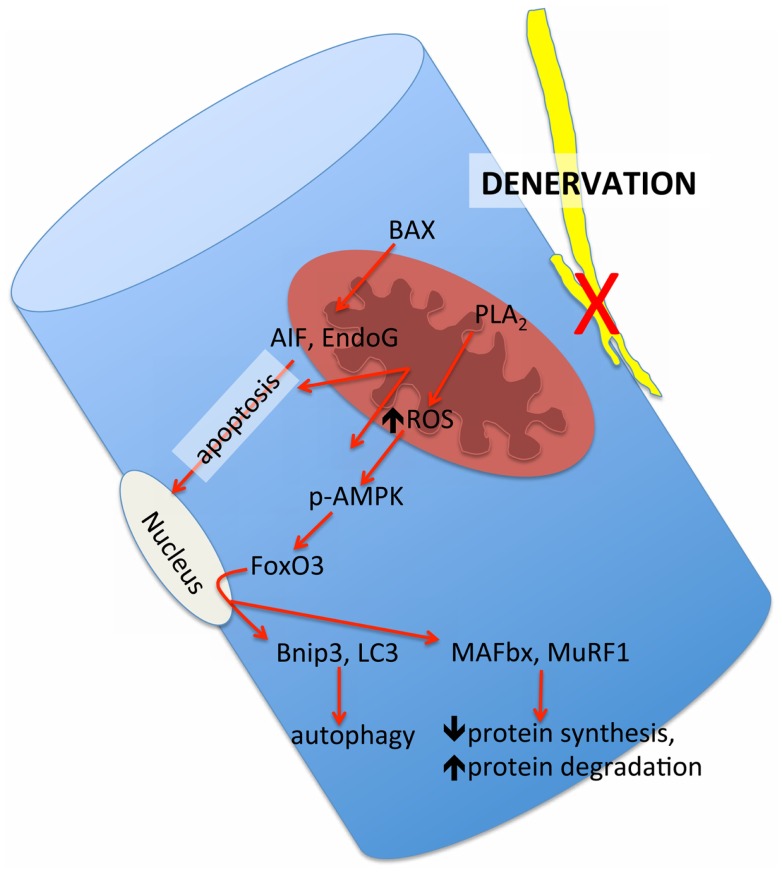
**Schematic representation of the mitochondrial-mediated atrophy pathways that have been observed to be up-regulated in aging muscle and their hypothesized link to the upstream influence of sporadic denervation in very advanced age where denervation becomes pronounced**. BAX = Bcl2-associated X protein, PLA2 = cytoplasmic phospholipase A2, AIF = apoptosis inducing factor; EndoG = endonuclease G, p-AMPK = phosphorylated adenosine monophosphate kinase, FoxO3 = forkhead box O3, BNIP3 = BCl2/adenovirus E1B 19 kDa protein-interacting protein 3, LC3 = microtubule associated protein light chain 3, MAFbx = muscle atrophy F-box, MuRF1 = muscle ring finger 1.

Although evidence of an accumulation of denervated myofibers in skeletal muscle at very advanced age in humans thus far is based only upon the morphological appearance of small angular fibers (Scelsi et al., 1980) that are typical of neuropathologies such as amyotrophic lateral sclerosis and polyneurophathy (Baloh et al., 2007), the presence of denervated myofibers in very old mouse (Wang et al., 2005) and rat muscle (Rowan et al., 2012) has been confirmed by probing *in situ* for a sodium channel isoform typical of denervation in adult muscle, NAV1.5 (Kallen et al., 1990; Yang et al., 1991). Importantly, these denervated myofibers also demonstrate a marked up-regulation of the ubiquitin ligases muscle atrophy F-box (MAFbx) and muscle ring finger 1 (MuRF1) (Rowan et al., 2012), and the up-regulation of these proteins in response to denervation is dependent upon release of mitochondrial lipid hydroperoxides that can be detected in some ROS assays (Bhattacharya et al., 2009). Thus, denervation can also recruit mitochondrial-mediated proteolytic pathways, raising the likelihood that at least some of the alterations in mitochondria seen in skeletal muscle at very advanced age are a secondary event to denervation rather than a primary organelle defect. A schematic representation of how we hypothesize that denervation may modulate mitochondrial pathways of atrophy in aging muscle is provided in Figure 8. Since understanding this issue is key to advancing to new therapeutic targets, future study of how sporadic denervation may modulate mitochondrial function across the continuum of aging muscle atrophy is clearly warranted to better define when intervening at the level of the mitochondrion is most appropriate.

## Conclusion

Our understanding of the impact of aging on mitochondrial function in aging skeletal muscle continues to evolve and has recently undergone significant revision. In particular, it now appears that mitochondrial functional alterations are more subtle than initially indicated, owing to an exaggeration of mitochondrial impact with aging when using isolated organelles (a common approach in early studies). Furthermore, whereas mitochondrial respiratory function and ROS emission changes are highly variable between muscles and are also largely attenuated by maintaining physical activity, a sensitization of the mitochondria to permeability transition seems to be a general property of aging muscles undergoing atrophy regardless of physical activity status. The mechanisms contributing to this mitochondrial functional alteration in aging muscle remains an area of intensive study. Presently, it seems that mtDNA-driven mechanisms are incompatible with the available evidence and instead impairment in mitophagy is appearing the more likely culprit based upon data from recent studies showing alterations in mitophagy signaling. In this respect, there is also emerging evidence that the aging *milieu* can itself depress mitochondrial respiratory function *in vivo*, even in the absence of an intrinsic organelle defect in mitochondria studied *ex vivo*. Furthermore, the likelihood that other extrinsic factors, such as denervation, may also be modulating mitochondrial function at very advanced and clinically relevant ages requires careful consideration of the most appropriate ages at which to target the mitochondrion in seeking more effective treatments for aging muscle. Based on current evidence, it is suggested that an increased susceptibility to permeability transition at ages preceding the most severe clinical impact of aging muscle atrophy (≤75 years) is an appropriate therapeutic target and should be pursued. However, at clinically relevant ages where an increased falls risk, mobility impairment, and physical frailty are more likely to result from aging muscle impact, it remains to be determined whether mitochondrial alterations are now largely secondary to denervation, rendering the mitochondrion a less attractive therapeutic target for the ≥ 80 years age group.

## Conflict of Interest Statement

The author declares that the research was conducted in the absence of any commercial or financial relationships that could be construed as a potential conflict of interest.
